# Diagnosis of Acute Cholecystitis Using T1 and T2 Mapping by Cardiac MRI

**DOI:** 10.1161/CIRCIMAGING.123.015605

**Published:** 2023-09-22

**Authors:** Gautam Sen, Kristopher Knott, Stefania Rosmini

**Affiliations:** King’s College Hospital, King’s College Hospital NHS Foundation Trust, Denmark Hill, London, United Kingdom (G.S., K.K., S.R.).; School of Cardiovascular Medicine and Sciences, King’s College London British Heart Foundation Centre of Excellence, United Kingdom (G.S.).

**Keywords:** cardiac MRI, cholecystitis, acute, MINOCA, T1 and T2 mapping

A 45-year-old woman was admitted to a tertiary hospital with acute chest pain radiating to the abdomen. She had a history of mild hypertension and an elevated body mass index of 32 kg/m^2^. She was an active smoker of 20 cigarettes a day and had an alcohol intake of 16 units/week. She had no significant family history of cardiovascular disease and did not take any regular medications. On admission to the emergency department, clinical examination was normal; however, serial 12-lead ECG showed transient inferior T-wave inversion. Routine bloods including full blood count, renal function, and liver function were normal. Troponin levels were mildly elevated and dynamic, and C-reactive protein was significantly elevated at 61 mg/L (normal, <1 mg/L). A computed tomography aortogram was performed, which showed no aortic dissection and flagged no other pathology of relevance.

In view of the cardiovascular risk factors, dynamic troponin, and ECG changes, she underwent invasive coronary angiography. This showed a nonobstructive lesion in the left anterior descending artery with no other major abnormalities (Video S1). She was treated medically for a presumptive diagnosis of myocardial infarction with nonobstructed coronary arteries (MINOCA) with aspirin and statin, and was referred for an inpatient cardiovascular magnetic resonance (CMR) for further assessment.

CMR at 1.5T showed normal biventricular size and function with normal left ventricular ejection fraction of 58% and no regional wall motion abnormalities. On T1 and T2 mapping, there was no evidence of edema or diffuse myocardial fibrosis. Late gadolinium enhancement revealed no myocardial infarction or focal fibrosis. Unexpectedly, on the T1 maps, an enlarged and distended gallbladder filled by an unusual fluid was noted raising the suspicion of an exudative process (Figure 1A; star: 1547 ms) with edematous walls (Figure 1A; yellow arrowhead: T1, 2388 ms; normal values for myocardium, 950–1100 ms) and multiple stones (Figure 1A, green arrows). Cine images confirmed multiple stones with one located in the cystic duct (Figure 1B, green arrow) with low T1 values (T1, 400 ms) suggesting a high cholesterol component. T2 mapping confirmed high values matching the T1 findings for the gallbladder and gallbladder walls (Figure 1C; black arrowhead: T2, 161 ms; normal values for myocardium, ≤48 ms) in keeping with edema. Overall, the findings raised the suspicion of acute cholecystitis.

**Figure 1.**
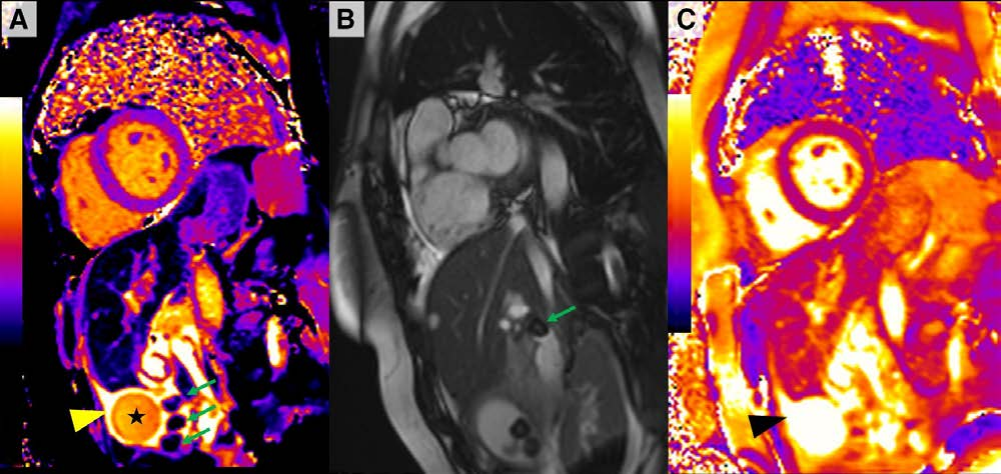
**Cardiac magnetic resonance. A**, Short-axis parametric modified look-locker inversion recovery T1 map showing an enlarged gallbladder filled by a fluid raising suspicion of an exudative process (black star: T1 map, 1547 ms) with edematous walls (yellow arrowhead: T1, 2388 ms) and multiple stones (green arrows) with low T1 values (T1, 400 ms) suggesting a high cholesterol component. **B**, Short-axis steady-state free-procession cine image showing multiple gallbladder stones with one located in the cystic duct (green arrow). **C**, Short-axis parametric T2 map showing high values in the gallbladder and gallbladder walls in keeping with fluid/edema (black arrowhead: T2, 161 ms).

She was reviewed by the gastrointestinal surgical team and proceeded to have further imaging including an ultrasound scan of the liver and magnetic resonance cholangiopancreatography, confirming an obstructing gallstone in the cystic duct with resulting gallbladder distension. The following day, she underwent urgent laparoscopic cholecystectomy with removal of a distended gallbladder (Figure 2). Histopathology of the gallbladder showed extensive mucosal ulceration and transmural congestion/inflammation in keeping with acute-on-chronic cholecystitis with 4 green stones, suggesting of being cholesterol rich. She was discharged home 2 days later and has been well since on cardiac and surgical follow-up.

**Figure 2.**
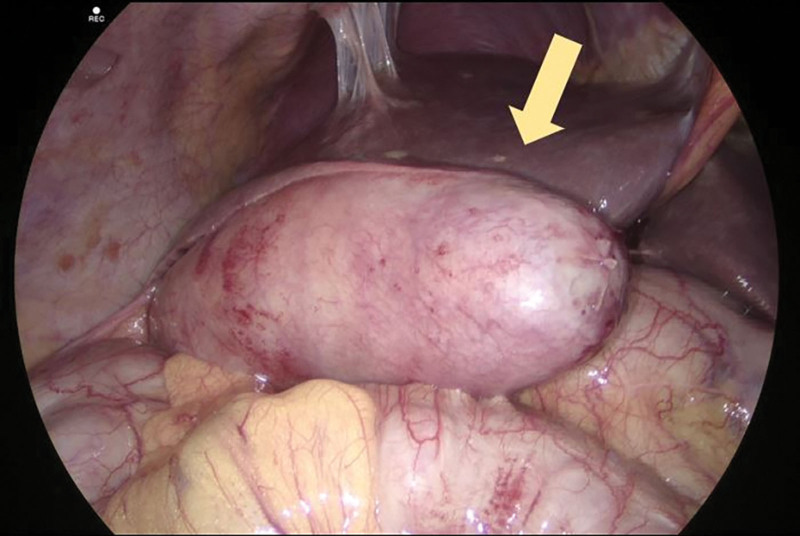
**Laparoscopic cholecystectomy.** Enlarged and inflamed gallbladder (yellow arrow) with multiple stones removed during an uncomplicated laparoscopic cholecystectomy (stones not shown).

CMR is recommended by contemporary clinical practice guidelines in patients with MINOCA to confirm the presence of an ischemic pattern and to exclude other nonischemic cardiac causes, having both a diagnostic and prognostic value.^1^ Furthermore, our case highlights the additional utility of CMR in the identification of extracardiac causes of chest pain.

Recent research has shown that incidental extracardiac findings are seen in up to 35% of CMR studies, with major extracardiac findings in up to 12% and extracardiac findings changing management in 1% of patients.^2^ Even though the majority of extracardiac findings are likely to be benign, this was not the case for our patient who would have been discharged by the cardiology team if the pathology was not identified. This would have resulted in a delay in the diagnosis of acute cholecystitis leading to worse clinical outcomes, including possible severe sepsis, gallbladder perforation, and presentation as acute abdominal syndrome.^3^

While T1 mapping is usually utilized for myocardial assessment, more recently, a role has been shown in the assessment of pleural and pericardial effusions.^4^ To the best of our knowledge, parametric mapping has not been previously shown to diagnose acute cholecystitis, highlighting the possible diverse application of the technique. Caution should be applied in the interpretation of the mapping images for extracardiac findings, and integration with other imaging modalities should be sought.

## ARTICLE INFORMATION

### Acknowledgments

The authors would like to thank Professor Ameet Patel and Francesca Forbes-Boulter for their help in this case.

### Sources of Funding

G. Sen is funded by the British Heart Foundation grant FS/CRTF/22/24368.

### Disclosures

None.

### Supplemental Material

Video S1

## Supplementary Material



## References

[1] Mileva N Paolisso P Gallinoro E Fabbricatore D Munhoz D Bergamaschi L Belmonte M Panayotov P Pizzi C Barbato E. Diagnostic and prognostic role of cardiac magnetic resonance in MINOCA: systematic review and meta-analysis. JACC Cardiovasc Imaging. 2023;16:376–389. doi: 10.1016/j.jcmg.2022.12.029 36889851 10.1016/j.jcmg.2022.12.029

[2] Rodrigues JC Lyen SM Loughborough W Amadu AM Baritussio A Dastidar AG Manghat NE Bucciarelli-Ducci C. Extra-cardiac findings in cardiovascular magnetic resonance: what the imaging cardiologist needs to know. J Cardiovasc Magn Reson. 2016;18:26. doi: 10.1186/s12968-016-0246-1 27156861 10.1186/s12968-016-0246-1 PMC4860770

[3] Gallaher JR Charles A. Acute cholecystitis: a review. JAMA. 2022;327:965–975. doi: 10.1001/jama.2022.2350 35258527 10.1001/jama.2022.2350

[4] Rosmini S Seraphim A Knott K Brown JT Knight DS Zaman S Cole G Sado D Captur G Gomes AC. Non-invasive characterization of pleural and pericardial effusions using T1 mapping by magnetic resonance imaging. Eur Heart J Cardiovasc Imaging. 2022;23:1117–1126. doi: 10.1093/ehjci/jeab128 34331054 10.1093/ehjci/jeab128 PMC9612798

