# Supplementary material for: Diagnosis of Acute Cholecystitis Using T1 and T2 Mapping by Cardiac MRI
Source: Circ Cardiovasc Imaging. 2023 Sep 22;16(10):e015605. doi: 10.1161/CIRCIMAGING.123.015605 (PMC10581435; doi:10.1161/CIRCIMAGING.123.015605)
Supplement: Supplementary file 1 [file hci-16-e015605-s001.pdf]

SUPPLEMENTAL MATERIAL

Video Legend

Video S1: Invasive coronary angiography Invasive coronary angiography showing a non-obstructive lesion in the left anterior descending artery with no other major abnormalities.
